# Nomogram for Preoperative Estimation of Cervical Lymph Node Metastasis Risk in Papillary Thyroid Microcarcinoma

**DOI:** 10.3389/fendo.2021.613974

**Published:** 2021-03-31

**Authors:** Jinxiao Sun, Qi Jiang, Xian Wang, Wenhua Liu, Xin Wang

**Affiliations:** ^1^ Department of Ultrasonography, Taihu Lake Cadre Sanatorium of Jiangsu Province, Wuxi, China; ^2^ Department of Ultrasonography, Affiliated Renmin Hospital, Jiangsu University, Zhenjiang, China; ^3^ Department of Ultrasonography, Yunyang People’s Hospital of Danyang, Danyang, China

**Keywords:** thyroid cancer, microcarcinoma, lymphatic metastasis, nomograms, ultrasound

## Abstract

**Objective:**

Accurate preoperative identification of cervical lymph node metastasis (CLNM) is essential for clinical management and established of different surgical protocol for patients with papillary thyroid microcarcinoma (PTMC). Herein, we aimed to develop an ultrasound (US) features and clinical characteristics-based nomogram for preoperative diagnosis of CLNM for PTMC.

**Method:**

Our study included 552 patients who were pathologically diagnosed with PTMC between January 2015 and June 2019. All patients underwent total thyroidectomy or lobectomy and divided into two groups: CLNM and non-CLNM. Univariate and multivariate analysis were performed to examine risk factors associated with CLNM. A nomogram comprising the prognostic model to predict the CLNM was established, and internal validation in the cohort was performed.

**Results:**

CLNM and non-CLNM were observed in 216(39.1%) and 336(60.9%) cases, respectively. Seven variables of clinical and US features as potential predictors including male sex (odd ratio [OR] = 1.974, 95% confidence interval [CI], 1.243-2.774; P =0.004), age < 45 years (OR = 4.621, 95% CI, 2.160-9.347; P < 0.001), US-reported CLN status (OR = 1.894, 95% CI, 0.754-3.347; P =0.005), multifocality (OR = 1.793, 95% CI, 0.774-2.649; P =0.007), tumor size ≥ 0.6cm (OR = 1.731, 95% CI,0.793-3.852; P =0.018), ETE (OR = 3.772, 95% CI, 1.752-8.441;P< 0.001) and microcalcification (OR = 2.316, 95% CI, 1.099-4.964; P < 0.001) were taken into account. The predictive nomogram was established by involving all the factors above used for preoperative prediction of CLNM in patients with PTCM. The nomogram model showed an AUC of 0.839 and an accuracy of 77.9% in predicting CLNM. Furthermore, the calibration curve demonstrated a strong consistency between nomogram and clinical findings in prediction CLNM for PTMC.

**Conclusions:**

The nomogram achieved promising results for predicting preoperative CLNM in PTMC by combining clinical and US risk factor. Our proposed prediction model is able to help determine an individual’s risk of CLNM in PTMC, thus facilitate reasonable therapy decision making.

## Introduction

The World Health Organization defines papillary thyroid microcarcinoma (PTMC) as a subset of papillary thyroid carcinoma (PTC), which is ≤1.0 cm at the greatest dimension (1). The incidence of PTC, particularly PTMC, has risen considerably across the world in the past few decades (2). Most of these tumors are not easy to identify clinically because they are not palpable. Thus, the discovery of PTMC can be attributed to the application of routine high-resolution ultrasonography (US), as well as other imaging techniques (3). Despite slow growth and good prognosis are usually observed, some PTMC are accompanied by high-risk features at the time of diagnosis, such as cervical lymph node metastasis (CLNM) and extrathyroidal extension (ETE). This is strongly linked to distant metastasis, high locoregional recurrence, and enhanced death risk (4, 5). Recent evidence indicates a 24-64% incidence of CLNM in PTMC, which usually affects the central neck compartment (6–10).

Patients with PTMC usually undergo central lymph node dissection (CLND) as a standard and necessary procedure when they are suspected of having lymph node involvement in the neck (1). Nevertheless, prophylactic CLND for PTMC is still controversial and uncertain, because prophylactic CLND is expected to enhance therapy-related morbidities like recurrent laryngeal nerve injury, chyle leak, brachial plexus palsy, phrenic nerve palsy, hypoparathyroidism (11). So that, identifying risk factors for CLNM in patients with PTMC before surgery can have a profound impact on the prognosis.

Many studies have reported on the preoperative clinicopathologic risk factors of CLNM for PTMC (3, 6). But the findings have been conflicting (12, 13), and therefore, there has been no agreement on the subject. In addition, only clinicopathologic features were incorporated into the studies (14, 15). As a result, their clinical application is rather limited. Ultrasound (US) examination is a widely applied method for assessing thyroid nodules and cervical lymph node (CLN). The technique has several advantages: radiation-free, convenience, noninvasiveness, inexpensiveness, and real-time. Different from previous studies, our study aimed to establish a simple nomogram based on clinical, haematological and US features to predict the risk of CLNM preoperatively in PTMC. This will enable clinicians to make better clinical decisions and thus improve patient outcomes.

## Materials and Methods

### Population

The approval to conduct this retrospective study was provided by the Affiliated Renmin hospital of Jiangsu University. Informed consent requirement was not applicable. Records of 594 patients with surgically confirmed PTMC from January 2015 to June 2019 were retrieved from the hospital’s database. All patients had thyroid US examination as part of the presurgical evaluation. After reviewing the records, we excluded 42 patients due to the following reasons: without CLND, history of thyroidectomy, thyroid treatment before US examination, poor-quality US images, distant metastasis, or incomplete medical records. Finally, we included 552 patients in this study.

### US Examination

**Figure 1:**
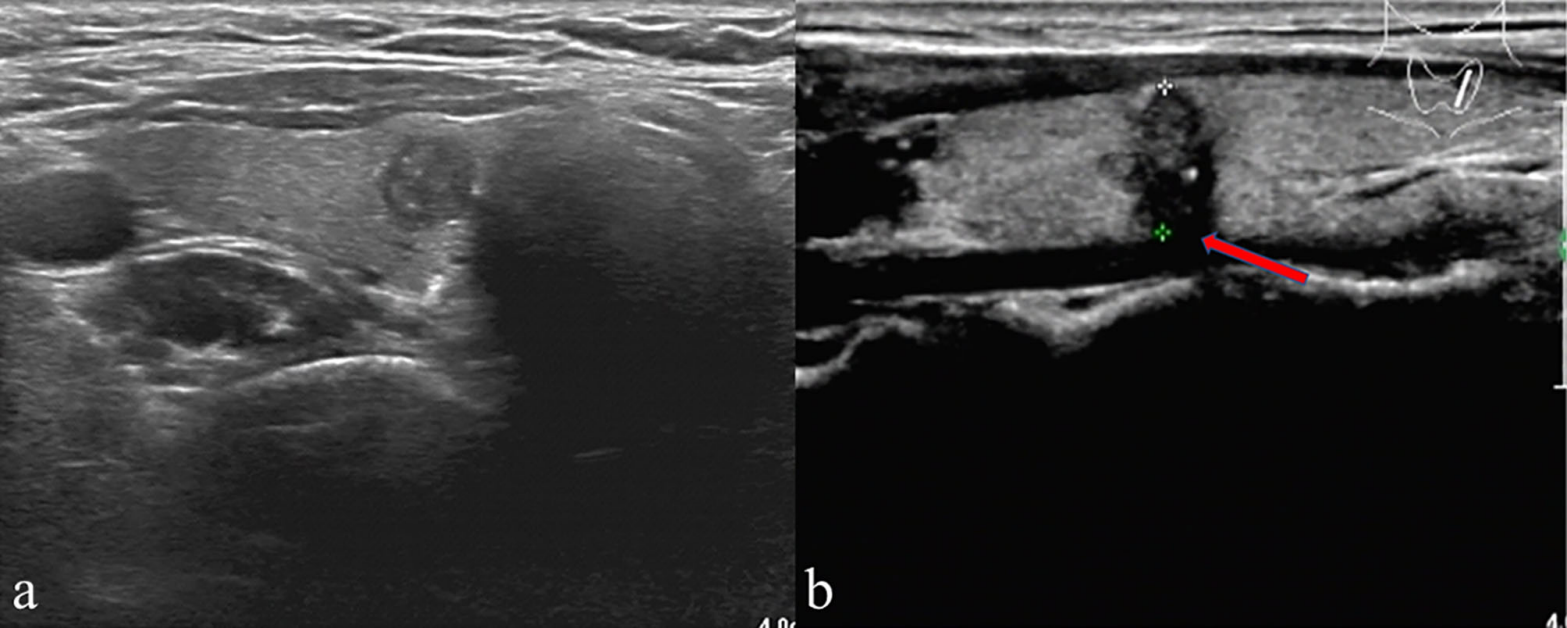
**(A)** A patient’s thyroid ultrasound showed punctated hyperecho in the nodule, indicating the presence of microcalcification. **(B)** A representative patient with ETE showed echogenic capsule line loss at the contact site of the lesion (red arrow) and microcalcification in the nodule.

US was performed using a high-resolution ultrasound scanner (iU22; Philips Healthcare, Eindhoven, the Netherlands) with a 5-12-MHz linear probe by an experienced radiologist. Then, the instrument was operated using a specific “Thyroid” program. Each patient adopted the supine position with the neck fully exposed, and the head lowered and moved slightly backward. Two experienced radiologists independently reviewed the US imaging features of every patient, both of them unaware of sample identity. If the radiologists disagreed, the final decision was made by consensus. Both cross-sectional and longitudinal examinations were conducted to determine the location. The following were the imaging features of each nodule: tumor size (the maximum diameter of the primary lesion), tumor margin, tumor shape, multifocality, bilaterality, aspect ratio (height divided by width on transverse views, A/T), microcalcification, tumor internal echo pattern, ETE and Hashimoto’s thyroiditis. Multifocality was considered in cases where one or both lobes exhibited two or more foci. Regarding multifocal PTMC, the largest, dominant tumor was first analyzed. For example, in multifocal cases, tumor size was classified based on the diameter of the largest tumor. The A/T was classified as ≤1 or > 1. Tumor margin was classified as smooth or ill-defined. Tumor shape was classified as either regular or irregular. Internal echo pattern was divided into heterogeneous or homogeneous. Microcalcifications were defined if their largest diameter was ≤ 2 mm (**Figure 1**). Based on the American Joint Committee on Cancer guidelines (16), ETE was defined by at least one of the following US features: thyroid capsule in contact with > 25% of the lesion perimeter, echogenic capsule line loss at the contact site of the lesion, tumor extension past the thyroid capsule, and invasion of the larynx, trachea, esophagus, recurrence laryngeal nerve, common carotid artery, mediastinal vessels, and subcutaneous soft tissues (**Figure 1**). The diagnosis of Hashimoto’s thyroiditis (HT) was made based on the US images and thyroid autoantibodies.

In the preoperative evaluation of CLNs, a suspicious lymph node exhibited the following characteristics: internal microcalcifications, hyperechoic change, round shape, vascularity, loss of hilar echogenicity, size > 5 mm, round shape, or necrosis.

The precision of operator-reported imaging features determined the diagnostic performance of our model; as such, we evaluated the interobserver reproducibility of the US features.

### Surgical Treatment

The protocol of thyroid surgery was established as per the guidelines of the American Thyroid Association (2015). Surgeries of ipsilateral lobectomy plus ipsilateral CLND were conducted as initial surgical therapy for PTMC patients with unilateral lesion. Total thyroidectomy plus ipsilateral or bilateral CLND was performed when PTMC lesion with ETE or multifocal carcinomas were restricted to a single lobe. When malignant lesions were found in both lobes of the thyroid, a total thyroidectomy plus a bilateral CLND was performed. Lateral lymph node dissection (LLND) was performed only in cases with clinically evident lateral neck lymph node metastasis (LLNM).

### Assessment of Clinical Variables

The clinical variables included age, sex, pre-operative TSH level, pre-operative TGAb level and pre-operative TPOAb level.

### Statistical Analysis

Clinical variables and US features were analyzed using univariate analysis. The R software (version 3.5.1) was used for data analysis. Data on continuous variables were expressed as mean ± standard deviation (SD). Categorical variables involved the number of cases. The importance of variables with CLNM was determined by binary logistic regression. A nomogram was established according to the results the binary logistic regression to evaluate risk of CLNM preoperatively. ROC was employed to quantify the discriminative capability of the nomogram by comparing nomogram-predicted versus the observed CLNM probability. Internally validated was performed by bootstraps with 1,000 resample to assess the accuracy of the constructed logistic regression model. A probability(p) value of 0.05 defined statistical significance.

## Results

### Demographics of PTMC Patients

In total, 552 patients were confirmed by pathological examination of surgical specimens, consisting of 450 (81.5%) women and 102 (18.6%) men. The patients had an average age of 45.5 ± 10.0 (22-75) years. Based on the pathological examination of surgical samples, 216 (39.1%) patients had CLNM, out of which 129 (23.4%) exhibited only central lymph node metastasis. In total, 63 (11.4%) patients had LLNM, whereas 24 (4.3%) patients had LLNM without central lymph node metastasis (leap metastasis). The subjective US-reported CLN status had a low accuracy of 0.677 for the whole cohort. The specificity was high (84.5%) but with low sensitivity (40.3%). A total of 127 patients were reported as LN negative but verified to have CLNM after the operation.

### Comparison Between Metastasis and No Metastasis Groups

The baseline clinical characteristics and US features with versus without CLNM are listed in **Table 1**. The age was significantly younger (p<0.001), and the CLNM group had considerably more male patients, relative to the non-CLNM group (27.8% vs. 19.3%, p=0.009). Based on US findings, the incidence of multifocality, bilaterality, and ETE was substantially higher in CLNM, relative to non-CLNM (p= 0.008, P=0.002, and p< 0.001, respectively). By contrast, the tumor size was relatively larger in CLNM than in non-CLNM (p< 0.001). Additionally, PTMC that had CLNM was more prone to microcalcifications, but no considerable difference was observed in HT rate, location, Pre-operative TSH, Pre-operative TGAb, Pre-operative TPOAb, tumor shape, tumor margin, A/T and internal echo between the two groups. Agreement was satisfactory between the two radiologists for the US features, with kappa coefficients between 0.78 and 0.89.

**Table 1:** Clinicopathological characteristics and US features associated with CLNM in PTMC patients.

Variables	CLNM(−)	CLNM(+)	P值
Sex			0.009
Male	65	60	
Female	271	156	
Age	51.0 ± 11.0	43.6 ± 11.1	<0.001
<45	252	105	<0.001
≥45~	84	111	
Location			0.657
Isthmus	12	5	
Upper	72	56	
Middle	162	109	
Lower	90	46	
HT			0.701
Negative	264	162	
Positive	72	54	
US-reported CLN status			<0.001
Negative	284	127	
Positive	52	89	
Multifocality			0.008
Negative	291	150	
Positive	45	66	
Bilaterality			0.002
Negative	306	193	
Positive	30	23	
Tumor size(cm)			0.025
<0.6	177	75	
≥0.6	159	141	
Margin			
Smooth	84	67	0.195
Ill-defined	252	149	
Shape			
Regular	47	37	0.676
Irregular	289	179	
Internal echo			
homogeneous	35	12	0.178
heterogeneous	301	204	
A/T			0.941
≤1	177	114	
>1	159	102	
Microcalcification			<0.001
Negative	201	66	
Positive	135	150	
ETE			<0.001
Negative	255	99	
Positive	81	117	
Pre-operative TSH (mU/L)			0.832
<2.5	219	135	
≥2.5	117	81	
Pre-operative TGAb (kU/L)			0.822
<1	210	141	
≥1	126	75	
Pre-operative TPOAb (kU/L)			0.630
<1	156	90	
≥1	180	126	

### Analysis of CLNM Risk Factors

Regarding univariate analysis, sex (P =0.002), age (P < 0.001), US-reported CLN status (P < 0.001), multifocality (P =0.001), bilaterality (P = 0.024), ETE (P < 0.001), tumor size (P=0.005) and microcalcifications (P < 0.001) are strongly linked to CLNM. Several variables were shown to be substantially related to CLNM based on multivariate logistic regression modeling. These included male sex (odd ratio [OR] = 1.974, 95% CI, 1.243-2.774; P =0.004), age < 45 years (OR = 4.621, 95% CI, 2.160-9.347; P < 0.001), US-reported CLN status (OR = 1.894, 95% CI, 0.754-3.347; P =0.005), multifocality (OR = 1.793, 95% CI, 0.774-2.649; P =0.007), tumor size ≥0.6cm (OR = 1.731, 95% CI,0.793-3.852; P =0.018), ETE (OR = 3.772, 95% CI, 1.752-8.441; P < 0.001) and microcalcifications (OR = 2.316, 95% CI, 1.099-4.964; P < 0.001) (**Table 2**).

**Table 2:** Univariate and multivariate analysis of 552 PTMC primary sites with clinical and US features for predicting CLNM.

Independent variable	Univariate		Multivariate	
	OR (95% CI)	P value	OR (95% CI)	P value
Age(years)				
≥45	1(reference)		1(reference)	
< 45	3.171(1.700–6.007)	<0.001	4.621 (2.160–9.347)	<0.001
Sex				
Female	1(reference)		1(reference)	
Male	2.374(1.243–3.774)	0.002	1.974(1.243–2.774)	0.004
US-reported CLN status				
Negative	1(reference)		1(reference)	
Positive	2.154 (0.995–3.547)	<0.001	1.894 (0.754–3.347)	0.005
Multifocality				
Negative	1(reference)		1(reference)	
Positive	2.845(1.369–6.061)	0.001	1.793 (0.774–2.649)	0.007
Bilaterality				
Negative	1(reference)		1(reference)	
Positive	1.372 (1.192–1.594)	0.024	1.074 (0.763-1.402)	0.763
Tumor size(cm)				
<0.6	1(reference)		1(reference)	
≥0.6	2.093(1.144–3.892)	0.005	1.731 (0.793–3.852)	0.018
Microcalcification				
Negative	1(reference)		1(reference)	
Positive	3.384(1.825–6.429)	<0.001	2.316 (1.099–4.964)	<0.001
ETE				
Negative	1(reference)		1(reference)	
Positive	3.721(1.988–7.092)	<0.001	3.772 (1.752–8.441)	<0.001

### Nomogram Construction

**Figure 2:**
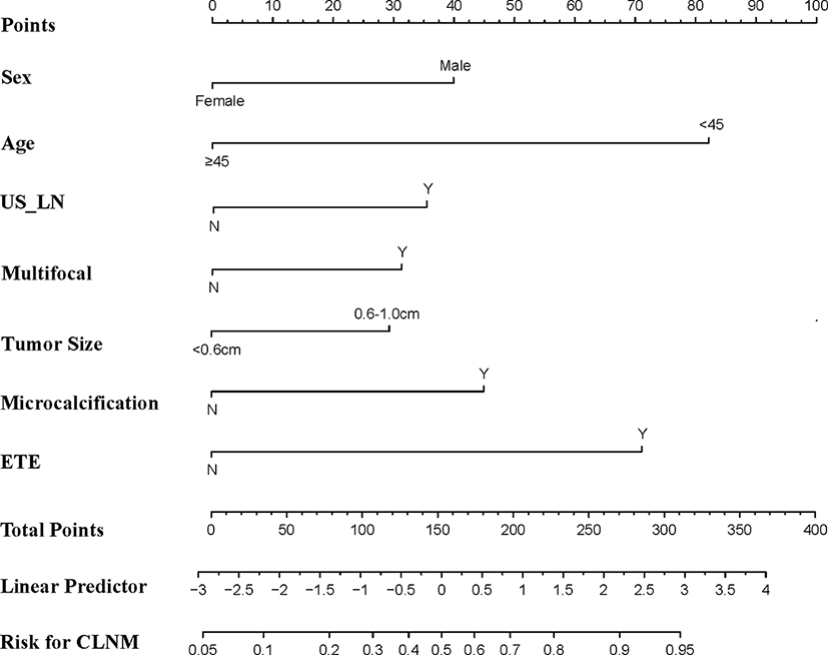
Nomogram for predicting CLNM in PTMC patients.

**Figure 3:**
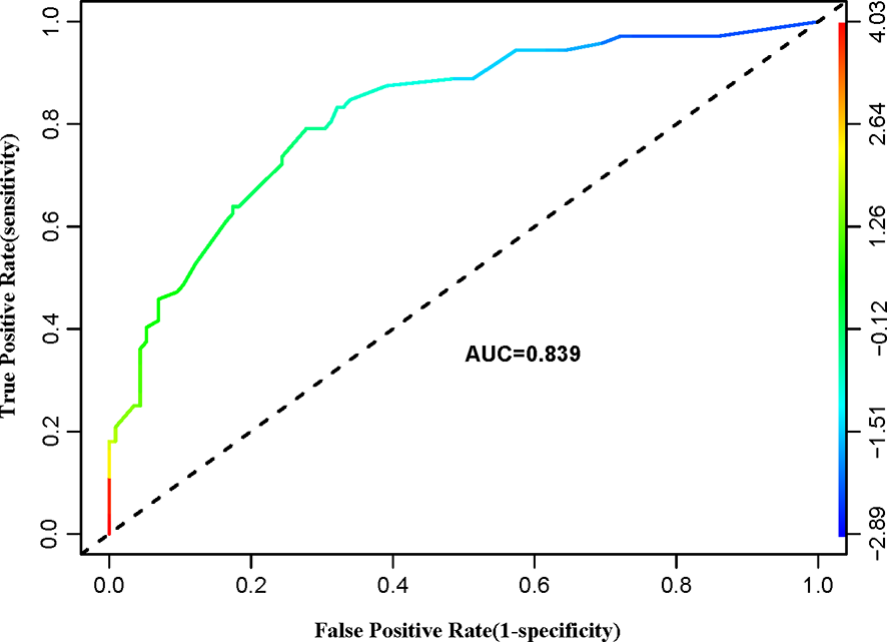
ROC curve analysis to predict CLNM in PTMC patients.

**Figure 4:**
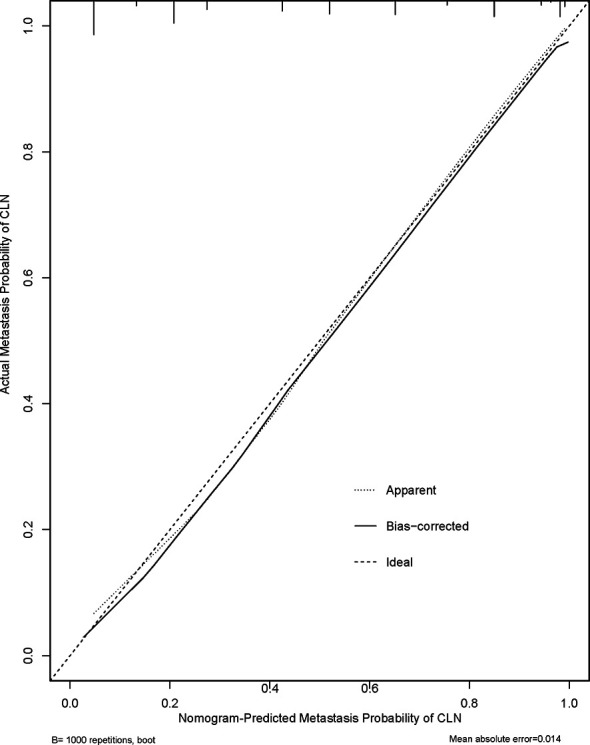
Calibration curves of the nomogram for predicting CLNM in PTMC patients.

**Figure 2** shows a nomogram created from important factors linked to CLNM. The nomogram contained seven risk factors (sex, age, US-reported CLN status, multifocality, tumor size, microcalcification, and ETE) to estimate the metastasis risk of CLNM for PTMC before surgery. Age yields the largest contribution to the prediction model, while ETE provides the next largest contribution. We assigned a score to every level within variables based on the point scale. Subsequently, we determined the risk of CLNM in each subject by summing up all total scores and identifying it on the total point scale. The predictive nomogram was also verified (**Figure 3**, AUC = 0.839,95%CI, 0.741-0.947) with sensitivity, specificity, and accuracy as 0.779, 0.715, and 0.830. The calibration plots exhibited an excellent consistency between the actual metastasis probability of CLN and predicted metastasis probability with additional 1000 bootstraps (**Figure 4**; Mean absolute error = 0.014).

## Discussion

To avoid overdiagnosis and the associated treatment, the guidelines of the American Thyroid Association (2015) recommend that active surveillance should be taken instead of surgical treatment for low-risk PTMC (1). The definition of low-risk PTMC is based on the absence of local invasion or distant metastasis, and convincing cytological evidence (1). Even in developed countries, the management of low-risk PTMC remains unclear (17, 18). This is because some PTMC progress during active surveillance, and are wrongly considered as low-risk (19). The study conducted by Choi et al. (20) showed that active surveillance could lead to poor disease outcomes because of clinically apparent lymph node metastasis. Most studies indicated that CLNM is a crucial recurrence risk factor, but is usually not detected in patients clinically (10, 21, 22). Therefore, identifying predictive factors that are linked to CLNM could guide appropriate surgical approaches for patients. Several previous studies have shown that CLNM prevalence ranged between 24% and 64% (8–10). Herein, the CLNM incidence in PTMC was 39.13%, and this was consistent with previous findings.

In our research, we established and internally validated a prediction model based on clinical and US features for predicting the probability of CLNM in PTMC patients. We have a major difference with other published studies (12, 23) because we evaluated non-invasive and preoperatively the individual probability of CLNM in PTMC. We analyzed the positive CLN involvement correlations between clinical characteristics, US features of primary thyroid lesions, and hemato-immunological parameters. Multivariate analysis revealed that male and young age (<45 years) were independent predictors for CLNM. This was consistent with the present results reported by others in patients with PTMC (24, 25). A higher basal metabolic rate in young male patients might enhance the proliferative and thus metastatic ability of tumor cells, and it will lead to more metastasis (26). Elevated levels of TSH, especially in conjunction with HT, are considered a risk factor for the development of thyroid malignancy and have been associated with a more advanced status of papillary thyroid carcinoma (27). However, the data regarding the impact of TSH and HT on CLNM in PTMC were inconsistent. Some studies have shown more lymph node metastasis in PTMC patients with HT (28), but others less (29). Some previous literatures focusing on PTMC patients found no association between the coexistence of HT and CLNM (30, 31). This is consistent with our study. Currently, ultrasonography is extensively applied in the evaluation of CLNM in PTMC patients, not only at the initial staging, but also in the course of subsequent surveillance after thyroidectomy (12). In our study, US features were the risk factors including US-reported CLN status, tumor size, ETE, multifocality and microcalcification. Tumors that exhibit certain features like a larger size, multifocality, and microcalcification on US examination were strongly linked to CLNM in our research, and similar findings have been reported previously (12, 32–34). Usually, ETE is a clinicopathological factor associated with CLNM (3, 12, 23, 28). But in our study, ETE was evaluated by US examination, which is determined by the purpose of our study. In addition, some studies have confirmed that US is satisfied with the evaluation of preoperative ETE (35), which provides the possibility for accurate prediction of CLNM before surgery. In our results concerning PTMC, univariate analysis indicated bilaterality as a risk factor for CLNM, however, multivariate analysis showed no statistical significance. The reason may be that multifocality and bilaterality were partially overlapped.

Our study built a predictive nomogram using the predictors identified in the multivariate regression model (**Figure 2**). The nomogram incorporates seven factors to generate a probability of a clinical event that is unique to an individual. This will finally assist clinicians in decision (36). The nomogram showed excellent discriminative capability (0.839, 95%CI, 0.741-0.947). Incorporating the clinical and US features into an easy-to-use nomogram enables preoperative individualized CLNM prediction. The nomogram established herein will help determine the existence of CLNM, which may avoid over-as well as under-treatment. Based on our results, we recommend that PTMC patients at a high risk of CLNM should undergo prophylactic lymph node dissection to prevent reoperations because of recurrence. Meanwhile, patients at low risk of CLNM should not receive prophylactic lymph node dissection because this may lead to unnecessary damage to the neck and possible surgical complications.

This retrospective observational study has some limitations. First, patients were enrolled from a single tertiary medical center, hence there was a potential for selection bias. Errors and biases tend to be higher in retrospective studies than in prospective studies. Second, for multifocal nodules, we only analyzed the largest one, when the information about other nodules was not available. Third, the performance of our nomogram depends on the operators with different levels of experience. The criteria used to evaluate the US signature were subjective. Nonetheless, there was shown to be an excellent interobserver agreement in our study. Finally, the sample size was not sufficiently large. As such, there is a need to conduct further studies involving a larger sample size and evaluation in external datasets to verify these findings.

In summary, we have built a predictive nomogram incorporating two clinical and five US features that can give a precise preoperative estimation of CLNM risk for each PTMC patients. However, further researches that involves larger samples sizes should be conducted to verify our findings.

## Data Availability Statement

The datasets used and/or analyzed during the current study are available from the corresponding author on reasonable request.

## Ethics Statement

Written informed consent was obtained from the individual(s) for the publication of any potentially identifiable images or data included in this article.

## Author Contributions

JS, XinW, QJ, XiaW, and WL contributed conception and design of the study. XinW supervised the project. QJ and WL organized the database. JS, XinW, QJ, and XiaW acquired, analyzed, and interpreted the patient date. JS and XinW wrote the first draft of the manuscript. All authors contributed to the article and approved the submitted version.

## Funding

This study was supported in part by a grant-in-aid for scientific research from the Jiangsu Provincial Commission of Health and Family Planning (Grant Nos. ZDXKC2016011), Wuxi Municipal Bureau on Science and Technology (Grant No. CMB41S1701) and Zhenjiang Science and Technology support Plan of Social Development Project (Grant No. SH2020046).

## Conflict of Interest

The authors declare that the research was conducted in the absence of any commercial or financial relationships that could be construed as a potential conflict of interest.
